# Effect of Probiotics Supplementation on Bone Mineral Content and Bone Mass Density

**DOI:** 10.1155/2014/595962

**Published:** 2014-01-22

**Authors:** Kolsoom Parvaneh, Rosita Jamaluddin, Golgis Karimi, Reza Erfani

**Affiliations:** ^1^Department of Nutrition and Dietetics, Faculty of Medicine and Health Sciences, University Putra Malaysia, 43400 Serdang, Selangor, Malaysia; ^2^Department of Orthopaedics and Traumatology, Faculty of Medicine, Universiti Kebengsaan Malaysia, 56000 Kuala Lumpur, Malaysia

## Abstract

A few studies in animals and a study in humans showed a positive effect of probiotic on bone metabolism and bone mass density. Most of the investigated bacteria were *Lactobacillus* and *Bifidobacterium* . The positive results of the probiotics were supported by the high content of dietary calcium and the high amounts of supplemented probiotics. Some of the principal mechanisms include (1) increasing mineral solubility due to production of short chain fatty acids; (2) producing phytase enzyme by bacteria to overcome the effect of mineral depressed by phytate; (3) reducing intestinal inflammation followed by increasing bone mass density; (4) hydrolysing glycoside bond food in the intestines by *Lactobacillus* and *Bifidobacteria*. These mechanisms lead to increase bioavailability of the minerals. In conclusion, probiotics showed potential effects on bone metabolism through different mechanisms with outstanding results in the animal model. The results also showed that postmenopausal women who suffered from low bone mass density are potential targets to consume probiotics for increasing mineral bioavailability including calcium and consequently increasing bone mass density.

## 1. Introduction

Probiotics literally means “for life” and the word is derived from Latin and Greek. However, the definition of probiotics has been varied since it was first coined several years ago [1]. Some of the probiotics are bacterial components comprising of normal human intestinal flora that produce end products of short chain fatty acids and lactate metabolism. These bacterial have potential clinical effects for the treatment or prevention of the intestinal and extra intestinal origin diseases [2, 3]. Most commonly investigated bacteria with health benefit are *Lactobacillus* and *Bifidobacteria*. These effects may be useful for reducing the risk of some diseases [3], such as fatty liver disease, inflammatory bowel disease (IBD), cardiovascular disease (CVD), and diabetes [4–7]. This implies that the intestinal bacteria play an important role in the immune system [8, 9]. Recent researches also indicated the beneficial effects of intestinal microbials on bone health.

One of the main bone diseases which is associated with aging and postmenopausal condition is osteoporosis. Currently, 75 million people in Europe, Japan, and the USA are affected by osteoporosis. In 2020, more than 50 percent of the United States population is estimated to suffer from low bone density [10]. One of the most serious problems among women over 50 years of age is hip fracture due to osteoporosis. The possibility of wrist, hip, or spine fracture is estimated to be parallel to the risk of heart disease (approximately 15%) [11].

Sufficient calcium intake has been reported to support bone growth and prevent bone loss during the aging process. Milk and milk products supply 75% of calcium needed by the body in western countries [12], but the consumption of milk has been failing over the past decade [13]. Some specialists believe that one of the effective treatments for osteoporosis is hormone replacement therapy (HRT). However, because of the side effects of HRT, this treatment is not generally accepted, due to low adhesion, unwillingness, and aversion by many women. Moreover, HRT over long durations of time may lead to increase the risk of some types of cancer [14].

Hence, alternative ways for preventing and treating osteoporosis are being developed and recommended by research scientists worldwide. Exercise, including walking and light running, have been reported to maintain bone mass density (BMD) [15]. Another potential method of fighting osteoporosis is probiotics consumption.

To the best of our knowledge, there is no review paper to determine the effect of probiotic on bone health or metabolism. The following review illustrates recent knowledge on the effects of probiotics on BMD as well as bone mineral content (BMC). The search carried out according to the Google scholar, PubMed, ISI Web of knowledge, Medline, and Cochrane Central Register of Controlled Trials (http://www.cochrane.org/). The terms were used for searching the literature including Probiotics, calcium absorption, BMD, BMC, bone loss, *Lactobacillus*, and *Bifidobacterium*.

## 2. Probiotics and Bones: Animal Studies 

Previous studies indicated that different strains of bacteria such as *Lactobacillus* and *Bifidobacterium* have the potential effect on increasing bone mass density on ovariectomized (OVX) rats and mice which can simulate postmenopausal conditions [16–19].

Chiang and Pan [18] used *Lactobacillus paracasei* (NTU 101) and *Lactobacillus plantarum* (NTU 102) (1 × 10^8^ CFU/ML) in OVX mice. They analyzed the right femur using computed tomography (CT) system, while the left femur was analyzed using scanning electron microscopy (SEM). The results showed that OVX mice fed with NTU 101 and NTU 102 fermented milk had higher trabecular number (Tb.N) compared to OVX and sham-ovariectomized (Sovx) as the control groups. In parallel with previous results, another study on male osteoporosis rats, but using different strain of bacteria, showed that* Lactobacillus helveticus* fermented milk increased BMD and BMC which were measured by using dual-energy X-ray absorptiometry (DEXA) [19].

A study on *Lactobacillus casei, Lactobacillus reuteri, *and* Lactobacillus gasseri* reported higher apparent calcium absorption in growing rats and 35% higher bone weight among the probiotic fed group compared to the control group [20]. Besides, Kim et al. [21] indicated that decreased level of BMD in OVX rats will be significantly improved by administrating *Lactobacillus casei 393* from fermented milk. They assessed the breaking force using a Texture Analyzer. They observed that bone strength of sham-ovariectomy (Sovx) group was higher than the Ovx group. However, after treatment in the Ovx group receiving fermented milk, the bone strength was not different from the Sovx group. No difference in body weight of rats was observed initially, but at the end surprisingly, the Sovx group had lower body weight as compared to the other groups. It was reported that ovariectomy caused an increase in dietary intake of rats and subsequently resulted in weight gain.

A current research showed a significant effect of *L. reuteri *6475 on bone health via decreasing tumor necrosis factor (TNF) levels and reducing bone resorption. The results showed increasing bone fracture, BMD, BMC, trabecular number and thickness, and decreasing trabecular space in both vertebral and femoral bones. In addition, body weight and fat pad weight showed a decreasing trend in the treated group [16]. Surprisingly, significant effects were observed only in treated healthy male mice not in females. Mutuş et al. [22], also worked on bacillus strain of *licheniformis *and *subtilis* on twenty-five one-year-old broiler chicks which were divided into the control and treatment groups. Diet in treatment group contained *Bacillus licheniformis *and *Bacillus subtilis. *After treatment, no significant difference was observed in the body weight and feed consumption between the two groups, but greater concentration of phosphorus and tibia ash, lateral and medial wall thickness of the tibiotarsi was observed in the group supplemented with probiotics compared to the control group fed with normal diet. While, the normal diet group had greater medullary canal diameter [22].

*Bifidobacterium longum* from fermented broccoli also showed a significant effect on bone health [17]. Four groups of male rats with different diets including normal diet and high cholesterol (HCD) for control groups and HCD + broccoli, HCD + *Bifidobacterium longum* from fermented broccoli diet for intervention groups were used. Significantly higher serum total cholesterol, higher potential antioxidant (PAO) serum level and 34% higher periodontal GSH/GSSG ratio was observed among intervention groups compared to the control groups. Beside a low number of TRAP-positive osteoclasts was found in the group fed with fermented broccoli compared to the control groups, while no significant differences were observed in body weight of rats between the groups. However, *Bifidobacterium longum *and yacon flour in another study have done by Rodrigues et al. [23] showed contradictory results from other previous studies. They reported no difference between intervention and control groups on femur thickness, length, and strength of fracture. The study involved thirty two male Wistar rats; divided into four groups, with 8 animals in each. Different groups of treatment receiving the following diets: control diet; yacon flour; standard diet + *Bifidobacterium longum*; and yacon flour + *Bifidobacterium longum*. They assessed daily food intake and weekly body weight to determine the efficiency coefficient of food (CEA) by weight gain/total food intake. They observed no differences in body weight, total food intake and feed efficiency after 28 days of treatment, while they showed that mineral content including calcium (Ca), phosphorus (P), and magnesium (Mg) was higher in animals fed with *B. longum* and *B. longum* with yacon flour.

Table 1 summarizes results of some of the animal studies on the effects of probiotics on bones.

## 3. Probiotics in Human Studies

Based on the knowledge, a study assessed the effects of probiotics on bone in twenty postmenopausal women with a mean age of 65 years and mean body mass index (BMI) of 26 [24]. The study was a double-blind randomized crossover study. Subjects comprised of two groups. The group consuming *Lactobacillus helveticus* fermented milk was compared to the control milk consumption group with a six-day washout. A day before starting the study, all subjects were given milk product (600 mg calcium) to avoid different amounts of calcium that may affect urine calcium collected in the morning. The effects of the consumed milk products were assessed by measuring the serum Parathyroid hormone (PTH), ionised calcium (iCa), Ca, P, and carboxyterminal telopeptide of type I collagen (ICTP) at 0, 1, 2, 4, 6, and 8 hours, and urinary calcium (U-Ca) and creatinine (U-Crea) at 0, 2, 4, 6 and 8 hours.

Effect of *Lactobacillus* from fermented milk confirmed the reduction of PTH followed by increased in serum calcium levels. In consequence reduced bone resorption in post-menopausal women. The ionized serum calcium, total calcium, phosphate, and urinary calcium were higher in the group that consumed *L. helveticus *fermented milk compared to the control group [24].

## 4. In Vitro Studies of Probiotics on Bone

Increasing BMD by *Lactobacillus helveticus* (LBK-16H) was also confirmed by an in vitro study [25]. In this study effects of compounds on osteoblasts and osteoclasts were measured by culturing bone marrow of mice derived from osteoblast and osteoclast precursor [26] with slight modifications [27]. Cells of bone marrow were obtained from the femora and tibia of 8–12-week-old female mice.

The results of calcium accumulation measured in osteoblast cultures found that *Lactobacillus helveticus* (LBK-16H) from fermented milk whey can increase osteoblast activity by 1.3-1.4 times with the 1 × 10^−5^, 1 × 10^−4^, and 1 × 10^−3^ solutions. However, no significant effect on bone formation was found in vitro osteoclast cultures [25].

## 5. Possible Mechanisms

Some possible mechanisms of probiotics on bones exit. One of the potential effects of probiotics on bone possibly occurs via synthesis of vitamins [28]. Vitamins like D, C, K, and folate are involved in metabolism of calcium and are necessarily for bone formation [29, 30]. Moreover, bacteria produce short chain fatty acids which decrease PTH followed by an increase in mineral absorption via their solubilisation [31].

In cereal based food minerals are surrounded by phytate. The availability of minerals such as zinc, iron, copper, and calcium are depressed by phytate [32]. Bacteria produce phytaze enzyme, which can release minerals depressed by phytate, resulting in increased availability of minerals including calcium [33]. Moreover, increasing the bioavailability of minerals also happens in some foods with estrogenic activity due to hydrolysis glycoside bonds of estrogenic food in the intestines by *Lactobacillus* and *Bifidobacteria* [18].

Some probiotics produce peptides that have biological functions on the body. These are called bioactive peptides. Bioactive peptides production varies depending on the kind of bacteria used [34]. *Lactobacillus helveticus *produces the proline-containing peptides isoleucyl-prolyl-proline (IPP) and valyl-prolyl-proline (VPP) which may induce greater availability of minerals. Furthermore, some of the peptides are not absorbed but could support release of minerals from insoluble ion and thus enhance mineral absorption. Another possible mechanism of IPP and VPP is through inhibition preventing the formation of Angiotensin II (Ang II) from Angiotensin I (Ang I) [24]. In in vitro study Ang II stimulated bone resorption [35] and may act as a vasoconstrictor in bone vasculature [36].

Another mechanism of *L. helveticus*-fermented milk could be by reducing PTH and raising calcium levels in the blood serum [24]. Decrease in bone loss by *L. helveticus*-fermented milk was confirmed with casein-o-phosphopeptides (CPP). CPP is produced during digestion of milk in the intestine. In a previous study, dietary CPP has been revealed to prevent osteoporosis in ovariectomized rats by increasing the bioavailability of calcium [21, 37]. Increased calcium absorption by some probiotic bacteria like *Lactobacillus salivarius* (UCC 118) was demonstrated in Caco-2 cells [38].

Oxidative stress stimulates osteoclast differentiation [39] and *Bifidobacterium longum*-fermented broccoli can improve periodontal antioxidant status by decreasing NF-*κ*B gene expression. On the other hand, during the progression of alveolar bone loss iNOS is an important factor for inflammatory response and *Bifidobacterium longum*-fermented broccoli in periodontal tissue downregulates iNOS gene expression. Thus, immune modulatory action and antioxidant effects have a role in the inhibition of osteoclast differentiation of alveolar bone surfaces.

A recent study showed another mechanism in which intake of a probiotic reduces intestinal inflammation and increases bone mass density, meaning that microbes in the gut have a significant effect on bone health [40]. *Lactobacillus reuteri 6475 *reduces proinflammatory cytokine levels systemically, which leads to increased bone volume fraction; similarly, it reduces the expression of proinflammatory cytokine in Jejunum and ileum. *Lactobacillus reuteri 6475 *affects bone health either through increased calcium uptake by increasing calcium solubility or by reducing epithelial cell inflammation which facilitate the transport or through production and transformation of estrogen like compounds that affect the intestinal epithelium or circulate in the blood and directly affect the bone cells [16].

In addition to metabolic differences, human and laboratory rodents such as rats have physiological, anatomical, and biochemical differences in the gastrointestinal (G.I.) tract. These differences can cause significant variation in nutrients or drug absorption from the oral route. Solubility, dissolution rates and transit times of nutrients or drug molecules can be modified by some physiological factors such as bile, pancreatic juice, and pH. Furthermore, enterohepatic circulation of nutrients and colonic delivery of formulation can be affected by microbial content of the GI tract. On the other hand, the gut microbiota can metabolize a wide range of chemicals which may affect the nutrients absorption. Approximately 400 different microorganisms are found in the GI tract. Although, upper GI tract in human and rabbits contain few organisms, large numbers are found in the stomach and upper intestine of other animals [41].

Previous studies on the effect of probiotics and bone have all confirmed that probiotics can increase BMD and BMC and help reduce osteoporosis by different ways. In the present review, we investigated the possible mechanisms of microbiota on bone health among animal (Table 2). Hence, the exact mechanism of bacteria on bone health among human is still unclear. This question deserves further studies.

## Figures and Tables

**Table 1:** Animal studies effect of probiotics on bone.

Probiotic strain	Duration	Possible effect on bone	Subject	Method	Author and year
*Lactobacillus reuteri 6475 *	4 weeks	(i) ↑Bone volume fraction (ii) ↑Bone mineral density (iii) ↑Bone mineral content (iv) ↑Trabecular number and thickness of distal femur (v) ↓Trabecular space (vi) ↑Vertebral trabecular bone volume	Healthy male mice	Micro-CT	McCabe et al. 2013 [16]
*Bifidobacterium longum, fermented broccoli *	12 weeks	↓Number of TRAP-positive osteoclasts	Male Wistar rats	Histologic measurements	Tomofuji et al. 2012 [17]
*Bifidobacterium Longum (ATCC 15707) *	28 days	(i) Bone weight, thickness, and length/NS (ii) Strength of fracture/NS (iii) ↑Ca, Mg bone content	Male Wistar rats	(i) Stainless-steel caliper (ii) Three-point Texture Analyzer (iii) Plasma emission spectrophotometer	Rodrigues et al. 2012 [23]
*Lactobacillus paracasei (NTU 101) and Lactobacillus plantarum (NTU102) *	8 weeks	(i) Femur BMD/NS (ii) Tb.Th/NS (iii) ↑Tb.N (iv) ↓Tb.sp	Ovariectomized mice	(i) CT system (ii) SkyScan	Chiang and Pan 2011 [18]
*Active Lactobacillus casei 393 *	6 weeks	(i) Femoral length/NS (ii) ↑Dry weight of femur (iii) ↑Ca and P in dry femur (iv) ↑BMD and BMC (v) ↑Bone strength	Ovariectomized Sprague-Dawley rats	(i) Vernier caliper (ii) Inductively coupled plasma-optical emission (iii) DEXA (iv) Three-point Texture Analyzer	Kim et al. 2009 [21]
*Bacillus licheniformis and Bacillus subtilis *	6 weeks	(i) ↑Medial and lateral wall thickness of tibiotarsi (ii) ↓Medullary canal diameter	Broiler chicks	(i) Dial caliper (ii) Subtracting the thickness of the medial and lateral walls from the diameter at the diaphysis	Mutuş et al. 2006 [22]
*Lactobacillus helveticus LBK-16H *	14 weeks	(i) ↑BMD and BMC (ii) ↑Femur weight	Male rats induced-osteoporosis aging	DEXA	Narva et al. 2004 [24]

NS: not significant.

CT: computed tomography.

TRAP: tartrate resistant acid phosphatase.

Ca: calcium.

Mg: magnesium.

BMD: bone mass density.

Tb.Th: trabecular thickness.

Tb.N: trabecular number.

Tb.sp: trabecular separation.

BMC: bone mineral content.

DEXA: dual-energy X-ray absorptiometry.

**Table 2:** Possible mechanisms of probiotics on bone.

Possible mechanisms	
Produce SCFA→↑PH→↑mineral absorption via their solubilisation→↓bone loss	
Produce phytase enzyme→release minerals depressed by phytate→↑availability of minerals→↓bone loss	
Hydrolysis glycoside bonds of estrogenic food→↑availability of minerals	
Produce IPP and VPP→↓formation Ang II from Ang I→↓bone resorption	
Produce antioxidant status→↓osteoclast differentiation→↓bone loss	
Reduces intestinal inflammation→↓proinflammatory cytokine levels→↓epithelial cell inflammation→↑calcium uptake→↑bone volume fraction	

SCFA: short chain fatty acid.

IPP: isoleucyl-prolyl-proline.

VPP: valyl-prolyl-proline.

ANG II: Angiotensin II.

ANG I: Angiotensin II.
